# Effects of a Hemsball-Based Program on Children and Adolescents: A Systematic Review

**DOI:** 10.3390/jfmk10020139

**Published:** 2025-04-21

**Authors:** Daniel González-Devesa, Nerea Blanco-Martínez, Carlos Ayán-Pérez

**Affiliations:** 1 Grupo de Investigación en Actividad Física, Educación, y Salud (GIAFES), Universidad Católica de Ávila, Canteros, 05005 Ávila, Spain; daniel.gonzalez@ucavila.es; 2 Well-Move Research Group, Galicia Sur Health Research Institute (IIS Galicia Sur), SERGAS-UVIGO, 36310 Vigo, Spain; n.blanco@uvigo.gal; 3 Departamento de Didácticas Especiáis, Universidade de Vigo, 36310 Vigo, Spain

**Keywords:** balance, coordination, motor skills, physical education, reaction time

## Abstract

**Objectives:** This systematic review aims to critically analyze the available evidence on the effects of hemsball interventions in children and adolescents. **Methods:** This study followed PRISMA guidelines for identifying, selecting, and analyzing investigations. Systematic searches were conducted in five electronic databases (Web of Science, SportDiscus, Google Scholar, PubMed, and Scopus) until 23 February 2025. Only intervention studies with a control group assessing the effects of hemsball in children and adolescents were included. The methodological quality of randomized controlled trials was assessed using the PEDro scale, while comparative studies were analyzed using the MINORS index. **Results:** Out of 138 initially identified studies, five met the inclusion criteria. The included studies evaluated the effects of hemsball on balance, coordination, agility, manual dexterity, attention, and lower limb strength. The findings indicated significant improvements in balance, coordination, and manual dexterity in groups that participated in hemsball compared to controls. However, no significant differences were found in agility and lower limb strength. **Conclusions:** Hemsball programs appear to be effective in enhancing motor and coordination skills in children and adolescents, particularly in populations with special educational needs. However, the heterogeneity of the studies and the lack of information on intervention intensity limit the strength of the conclusions.

## 1. Introduction

Participation in physical activities during childhood and adolescence provides multiple benefits that go beyond improving physical, mental, and social health [1]. Regular physical activity at these stages has been shown to positively contribute to academic performance [2], cognitive function and development [3], better physical fitness and body composition [4], as well as the development of social skills, the promotion of values, and personal growth [5].

Despite these positive effects, the prevalence of physical inactivity among children and adolescents remains alarming. Approximately 80% of this population does not meet the activity recommendations set by the World Health Organization [6]. This phenomenon can be attributed to multiple factors, including a lack of motivation, limited sports-related knowledge, and the barriers children and adolescents face in their daily environment [7,8]. These barriers include time constraints due to academic or personal responsibilities, lack of suitable equipment, high costs associated with sports participation, and limited access to nearby sports facilities. Additionally, many children perceive themselves as physically weaker than others, find physical activity too exhausting, or face health-related limitations that further reduce their willingness to engage in exercise [7,8]. The scientific literature indicates that motivation is one of the main determinants of physical activity participation, as it directly influences both its initiation and maintenance [9].

In these circumstances, school physical education serves as a key space for promoting physical activity, as it provides equal opportunities and facilitates the inclusion of all students, including those with specific needs [1,10]. Physical education teachers play a fundamental role in encouraging physical activity through the selection of appropriate content and the design of engaging activities [11]. Additionally, they can create a positive motivational climate that fosters sports participation and enhances adherence to physical activity [12]. This is especially relevant for children and adolescents with functional diversity, who face additional barriers that hinder their participation in sports [13].

Therefore, physical education professionals face the challenge of implementing strategies that foster student motivation and minimize the barriers limiting their participation in physical activity. One of the most effective strategies is the incorporation of alternative sports, which offer innovative, engaging, and accessible proposals that encourage active involvement and the participation of all students [14,15]. These games serve as useful tools to promote cooperative work, support physical and motor development, and enhance creativity and personal autonomy [16]. According to some authors, an approach centered on allowing each student to progress at their own pace helps increase both learning satisfaction and commitment to physical activity [17,18].

In this regard, hemsball emerges as an innovative option within alternative sports. Hemsball is a game originating from Turkey, played on hard, flat surfaces. Players throw a special ball with the goal of making it bounce and pass through a hoop, following specific rules of precision and control [19]. This inclusive sport allows for individualized adaptations for each player [20] and is characterized by requiring a good level of manual dexterity and fine motor skills during shooting, as well as eye–hand–foot coordination, balance, and reaction speed during the game [21,22]. Its implementation in the school setting not only provides a novel alternative to promote physical activity but can also contribute to the overall development of students [23]. Moreover, hemsball stands out as a promising alternative to overcome many of the main barriers that hinder physical activity among children and adolescents. It can be played with minimal equipment, in small indoor spaces, school gyms, or even public parks. It does not require high levels of physical strength or endurance, which facilitates the participation of a broad range of students. Furthermore, the pace of the game can be adjusted to the participants’ abilities, allowing the active inclusion of students with different physical conditions or health problems.

Scientific evidence has demonstrated the positive effects of this game on various groups. In women with mild intellectual disability, improvements have been observed in cardiovascular parameters, such as resting heart rate, and in anthropometric indicators, such as body mass index [24]. Likewise, in children with intellectual disability and developmental coordination disorder, the combination of hemsball training and physiotherapy has shown positive effects on postural control and balance, both static and dynamic [25]. This aspect makes the game of hemsball an interesting strategy for children with specific needs, due to the difficulties they face in participating at school compared to their typically developing peers [26].

In this context, physical education professionals need the best available scientific evidence on the benefits of practicing hemsball for the motor and overall development of students. This can be achieved through systematic reviews that critically assess the existing research on the topic. To the authors’ knowledge, no systematic review has been conducted on this subject to date. Therefore, the aim of this review is to synthesize and critically analyze the available scientific evidence on the effects of hemsball interventions in children and adolescents.

## 2. Materials and Methods

This systematic review was conducted following the Preferred Reporting Items for Systematic Reviews and Meta-Analyses (PRISMA) guidelines [27]. This review was registered with the Open Science Framework (OSF), https://doi.org/10.17605/OSF.IO/3YX5E.

### 2.1. Search Strategy

Systematic searches were performed in five electronic databases (Web of Science, SportDiscuss, Google Scholar, PubMed, and Scopus) from their inception until 23 February 2025. The keyword “hemsball” was searched across all five databases.

### 2.2. Eligibility Criteria

Only randomized controlled trials (RCTs) or comparative studies reporting results on the effects of hemsball interventions in children and/or adolescents were eligible for inclusion in this review. This is because these designs provide the highest level of evidence. Studies were excluded if (a) the research lacked a control or comparison group; (b) hemsball was combined with other exercises or therapies; (c) the intervention consisted of a single session; or (d) the full text of the study was not available. No studies were excluded based on language.

### 2.3. Study Selection

Two authors independently reviewed titles and abstracts of the identified studies to assess their suitability. This process was conducted using Rayyan software 1.5.0. (QCRI, Doha, Qatar), which enabled blinded screening by both reviewers [28]. After independent screening, the authors compared their results until consensus was reached. Subsequently, full texts of all potentially relevant studies were obtained.

### 2.4. Data Extraction

From each article, data were extracted regarding the title, authors, year of publication, country, study design, participant characteristics, hemsball interventions, analyzed variables, and main results. One researcher conducted the data extraction, and a second researcher verified its accuracy.

### 2.5. Quality Assessment

The methodological quality of each RCT was obtained from the Physiotherapy Evidence Database (PEDro). If a trial was not listed in the database, two authors independently assessed its quality, resolving discrepancies through consensus. Quality classification was based on the following cutoff points: excellent (9–10), good (6–8), acceptable (4–5), and poor (<3) [29]. The methodological quality of comparative studies was evaluated using the Methodological Index for Non-Randomized Studies (MINORS) [30]. This instrument includes 12 items corresponding to 12 quality criteria for comparative studies. Each item is scored as 0 (not reported), 1 (reported but inadequate), or 2 (reported and adequate). The ideal total score for comparative studies is 24. One author applied the MINORS scale, and another author verified the assessment. In case of disagreement, a third author was consulted. Quality was considered high if the total score was 17 or higher, while a score below 17 was considered low quality [31].

## 3. Results

**Figure 1:**
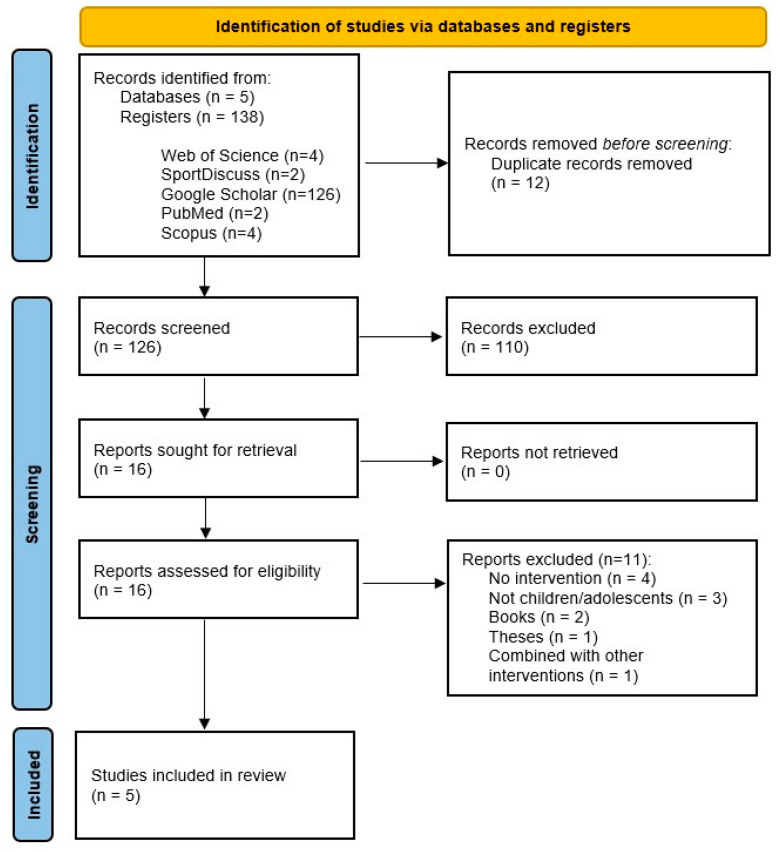
PRISMA (Preferred Reporting Items for Systematic Reviews and Meta-Analyses) study flow diagram.

From an initial pool of 138 references, 12 duplicates identified across the various databases were excluded. Ultimately, 16 studies were selected for full-text screening, of which 5 fulfilled the established eligibility criteria (Figure 1).

### 3.1. Study Design and Samples

Of the five included studies, three were RCTs [21,32,33], and two were comparative studies [22,34]. A total of 206 participants were included. Sample sizes ranged from 20 [32] to 80 participants [33]. Participants’ ages varied between 7 and 17 years; however, only three studies reported participants’ sex [22,32,33]. The participant characteristics were quite heterogeneous: two studies included children and adolescents with hearing loss [22,32], one study focused on adolescents actively playing on boccia teams [34], another included 80 primary school students [33], and one study included 50 adolescents with mild to moderate intellectual disabilities [21]. Table 1 provides a summary of their characteristics.

### 3.2. Characteristics of Interventions

The duration of the interventions included in this review ranged from 8 weeks [33,34] to 12 weeks [21]. Sessions lasted 60 min, with a frequency of three days per week across all studies. It is noteworthy that none of the five included studies reported information regarding intervention intensity. Additionally, none of the studies analyzed reported drop-outs or adverse effects associated with hemsball interventions. Control groups did not receive any additional training in any of the studies, except in the study by Şimşek et al. [34], where participants in the comparison group continued their usual boccia training.

### 3.3. Main Results

#### 3.3.1. Balance

Four studies analyzed the impact of hemsball programs on participants’ balance [21,32,33,34]. All found a significant improvement in balance post-intervention in groups practicing hemsball. Likewise, all four studies reported greater improvements in participants who practiced hemsball compared to control groups.

#### 3.3.2. Coordination

The two studies that included coordination as a primary variable demonstrated that hemsball programs improved eye–hand coordination [32], upper limb coordination, and bilateral coordination [21]. Similarly, Işık and Zorba [21] reported that a 12-week hemsball program significantly improved upper limb and bilateral coordination compared to the control group in adolescents with mild to moderate intellectual disabilities.

#### 3.3.3. Fine Motor Skills and Manual Dexterity

Işık and Kılıç [22] conducted a comparative study to analyze the effects of a hemsball program on fine motor precision, fine motor integration, and manual dexterity in children with hearing loss. Results showed significant improvements in the experimental group post-intervention for fine motor precision, fine motor integration, and manual dexterity. Furthermore, comparing both groups, the hemsball group showed superior scores in all these skills compared to the control group.

#### 3.3.4. Agility

The only study [33] examining the effects of an 8-week hemsball program on agility in primary school students found no significant differences post-intervention.

#### 3.3.5. Lower Limb Strength

The single study [33] examining the effects of an 8-week hemsball program on lower limb strength observed no significant differences post-intervention.

#### 3.3.6. Attention and Reaction Time

Only Işık and Kılıç [32] examined the effects of a hemsball program on attention and reaction time, reporting significant within-group improvements in both variables after ten weeks.

When comparing the hemsball group to the control group, which only attended physical education classes, a significantly greater improvement in attention was observed in the hemsball group after intervention. However, no significant differences were found between the groups in reaction time.

### 3.4. Quality Assessment Results

There was agreement in four out of the five studies evaluated; in one case, a discrepancy was resolved through consensus.

The methodological quality of reviewed RCTs was considered “good” (Table 2). Among the most relevant methodological issues not adequately addressed were participant, researcher, and assessor blinding, as well as concealed participant allocation to intervention conditions.

Of the two comparative studies analyzed, the study conducted by Şimşek et al. [34] was classified as high quality, whereas the study by Işık and Kılıç [22] was classified as low quality (Table 3). Among the methodological aspects most frequently identified as inadequate were an appropriate follow-up period consistent with study objectives and the prospective calculation of sample size.

## 4. Discussion

This systematic review aimed to evaluate and critically analyze the available evidence on the effects of hemsball interventions in children and adolescents. All but one of the studies showed good quality, which increases the consistency of the results. It is noteworthy that most of the analyzed studies focused on populations with disabilities, highlighting the potential of hemsball as an inclusive activity. The findings may help professionals in physical education and movement sciences consider hemsball as an effective activity for improving balance, coordination, and manual dexterity.

Among the different motor skills, static and dynamic balance has been the most studied variable in four investigations. During childhood, balance ability evolves as the child develops [35], reaching a level similar to that of adulthood around the ages of 7–8 years [36]. The within-group results show a positive impact of hemsball practice on balance, a finding that aligns with reports from other authors in children with developmental coordination disorder [25]. This could be due to the fact that participation in physical activities is an effective strategy for improving this ability [37]. Additionally, all studies indicated that hemsball provides greater benefits for balance compared to boccia, conventional physical activity classes, or the absence of changes in routine. This effect could be explained by the specific demands of hemsball, which require constant postural adjustments due to the need to maintain stability in a standing position while performing dynamic throwing and catching movements [38]. In contrast, boccia is played mostly in a seated position with lower postural demands [39], or conventional physical activity classes, where tasks may be more predictable and structured [40].

Coordination is a fundamental motor skill that develops progressively during childhood and adolescence, allowing for the efficient integration of movements in daily and sports activities [41]. The two studies that included coordination as a primary variable demonstrated that practicing hemsball significantly improves hand–eye coordination, upper limb coordination, and bilateral coordination. These findings align with those reported by Balayi et al. [25] in children with developmental coordination disorder. The observed improvement could be attributed to the specific characteristics of hemsball, which require constant synchronization between visual perception and motor response, thereby enhancing hand–eye coordination [38]. Additionally, performing simultaneous and alternating movements with both hands, along with the need to adjust posture in response to the trajectory of the ball and the opponent’s actions, may contribute to improvements in upper limb coordination and bilateral coordination. Furthermore, the studies indicated that hemsball provides greater benefits for coordination compared to other activities. This may be because, as Biino et al. [42] suggested, structured sports activities positively impact coordination, with early specialization depending on the sport practiced. This reinforces the importance of activities like hemsball, which involve fast and precise movements, adaptation to real-time stimuli, and decision-making under uncertain conditions, thus promoting the development of coordination.

The available evidence on agility (*p* > 0.05) and lower limb strength (*p* = 0.677) has not demonstrated significant benefits from practicing hemsball. This could be explained by the relatively short duration of the programs, which lasted 8 weeks and included a 4-week progressive instruction period, as well as the possible insufficiency of intensity to induce changes in these variables. However, other school-based games, such as jump rope or tag, have shown benefits in agility and lower limb strength parameters within the school environment [43,44].

In relation to attention, although improvements were observed within the group, no differences were found compared to the control group. However, other studies have shown that participation in ball games or activities is beneficial for the development of cognitive functions, including attention [45]. As for reaction time, although improvements were observed within the group, no significant differences were found compared to the control group (*p* = 0.255). This could be explained by the nature of the activity, as while hemsball involves quick responses to visual and motor stimuli, the intensity and specificity of the training may not have been sufficient to generate substantial improvements in this variable. Previous studies have shown that activities with higher demands on processing speed and quick directional changes, such as racquet sports [46], are more effective. Further studies are needed to analyze this variable, as both in the school population and in adult athletes, it has been observed that people with hearing loss may have shorter reaction times compared to those without disability [47,48].

Despite the originality of this review, certain limitations should be considered. First, the number of included studies was limited. Second, the studies varied in terms of populations, interventions, and outcome measures. For these reasons, a meta-analysis was not considered appropriate. Third, qualitative research was not considered, as the focus of this review was on quantitative data derived from hemsball interventions. Lastly, the exclusion of grey literature may have restricted the scope of the analyzed studies, potentially omitting relevant information.

## 5. Conclusions

The findings of this review suggest that hemsball programs can effectively improve balance, coordination, and manual dexterity in children and adolescents, especially in populations with special educational needs, while its effects on strength and agility are not evident. Therefore, hemsball programs may serve as an effective intervention to enhance motor and coordination skills in this group, and physical education professionals should consider incorporating them into their curricula. Physical education, occupational therapists, and other health professionals working with children who have motor difficulties may consider incorporating hemsball activities into their training sessions or rehabilitation programs as a playful and engaging way to promote motor control development. Furthermore, the specific features of hemsball may support the improvement of bilateral coordination and hand–eye coordination in both clinical and educational settings. However, further research is required to validate these findings. Given the limited number of studies, future comparative research is essential to assess the effectiveness of hemsball in improving other critical aspects of motor skills.

## Figures and Tables

**Table 1:** Descriptive characteristics of included studies.

First Author (Year), Design	Sample	Intervention	Measurements	Results (*p* < 0.05)
Şimşek et al. (2024) [34] Design: Comparative	Participants (n): 30 who actively play in boccia teams (EG = 15; CG = 15) Age, years (Mean ± SD): EG = 12.93 ± 0.96 CG = 12.86 ± 0.91	Duration: 8 weeks EG Type: Hemsball Program Activities: They completed the hemsball program after their boccia training sessions. It included warm-up exercises, basic hemsball techniques and tactics, throwing and receiving techniques, as well as competition and training games. Volume: 60 min/session Frequency: 3 days/week Intensity: NR CG—They continued with their boccia training	Balance (1) Static (stabilometric platform) (2) Dynamic (Libra, EasyTech)	Intra-group EG ↑ Static equilibrium Inter-group Improvement in static balance EG > CG Improvement in dynamic balance EG > CG
Işık and Kılıç (2022) [32] Design: RCT	Participants (n): 20 with hearing loss; 11F + 9M (EG = 10; CG = 10) Age, years (average): EG = 13.6 CG = 14.1	Duration: 10 weeks EG Type: Hemsball Program Activities: Included warm-up exercises with throwing, rebounding and catching, followed by specific exercises such as shooting hoops, target wall, passing between teammates and hemsball juggling. Ended with stretching. Volume: 60 min/session Frequency: 3 days/week Intensity: NR CG—They only attended physical education classes.	Balance (Bruininks–Oseretsky balance sub-scale) Attention (BAT) Reaction time (Simple Reaction Time Test) Eye–hand coordination (Alternate Hand Wall Toss Test)	Intra-group EG ↑ Balance ↑ Attention ↑ Reaction time (dominant hand) ↑ Eye–hand coordination Inter-group Attention EG > CG Balance EG > CG
Işık and Kılıç (2021) [22] Design: Comparative	Participants (n): 26 with hearing loss; 12F + 14M (EG = 13; CG = 13) Age, years (range): 7–11	Duration: 10 weeks EG Type: Hemsball Program Activities: Included exercises to improve fine motor skills and manual dexterity, based on different hemsball throwing techniques Volume: 60 min/session Frequency: 3 days/week Intensity: NR CG—Did not receive any training	Fine motor skills and manual dexterity (Bruininks–Oseretsky Motor Proficiency Test)	Intra-group EG ↑ Fine motor precision ↑ Fine motor integration ↑ Manual dexterity Inter-group Fine motor precision EG > CG Fine motor integration EG > CG Manual dexterity EG > CG
Işık and Zorba (2020) [21] Design: RCT	Participants (n): 50 with mild and moderate intellectual disabilities (EG = 25; CG = 25) Age, years (Mean ± SD): 13.85 ± 0.93	Duration: 12 weeks EG Type: Hemsball Program Activities: The session included warm-up (10 min), ball exercises (45 min) for precision and coordination, and cool-down (5 min) with walking and stretching. Volume: 60 min/session Frequency: 3 days/week Intensity: NR CG—Did not receive any training	Balance (Bruininks–Oseretsky balance sub-scale) Bilateral coordination (Bruininks–Oseretsky sub-scale) Upper limb coordination (Bruininks–Oseretsky sub-scale)	Intra-group EG ↑ Balance ↑ Bilateral coordination ↑ Upper limb coordination Inter-group EG > CG balance EG > CG bilateral coordination Upper limb coordination EG > CG
Sever et al. (2016) [33] Design: RCT	Participants (n): 80 elementary students, 40F + 40M (GE = 50; CG = 30) Age, years (Mean ± SD): EG: 8.82 ± 1.14 CG: 9.00 ± 1.19	Duration: 8 weeks EG Type: Hemsball Program Activities: They were progressively instructed in technical techniques, such as throwing and standing positions. In the last 4 weeks, organized matches and games were held. Volume: 60 min/session Frequency: 3 days/week Intensity: NR CG—Did not receive any training	Balance (Stork balance test) Agility (Reactive agility test) Lower extremity strength (Vertical jump test)	Intra-group EG ↑ Balance Inter-group Balance EG > CG

BAT = Bourdon Attention Test; RCT = Randomized controlled trial; F = Female; CG = Control group; EG = Experimental group; M = Male; NR = Not reported.

**Table 2:** Quality Assessment of Randomized Controlled Trials.

	Items												Quality Rating
Authors (Year)	1	2	3	4	5	6	7	8	9	10	11	Score	
Işık and Kılıç (2022) [32]	Y	1	0	1	0	0	0	1	1	1	1	6/10	Good
Işık and Zorba (2020) [21]	Y	1	0	1	0	0	0	1	1	1	1	6/10	Good
Sever et al. (2016) [33]	Y	1	0	1	0	0	0	1	1	1	1	6/10	Good

Eligibility criteria item does not contribute to total score. Items: (1) eligibility criteria; (2) randomization; (3) concealed allocation; (4) similarity at baseline; (5) subjects blinding; (6) therapists blinding; (7) assessors blinding; (8) one key outcome measured in >85% of subjects; (9) intention-to-treat analysis; (10) between-group statistical results for one key outcome; (11) measures of variability and point measures for one key outcome.

**Table 3:** Risk of bias assessed by MINORS index.

First Author (Year)	1	2	3	4	5	6	7	8	9	10	11	12	Points
Şimşek et al. (2024) [34]	2	1	1	2	1	0	2	0	2	2	2	2	17/24
Işık and Kılıç (2021) [22]	2	1	1	2	1	0	2	0	2	2	1	2	16/24

Items: (1) clearly stated aim; (2) inclusion of consecutive patients; (3) prospective collection of data; (4) endpoints appropriate to the aim of the study; (5) unbiased assessment of the study endpoint; (6) follow-up period appropriate to the aim of the study; (7) loss to follow up less than 5%; (8) prospective calculation of the study size; (9) adequate control group; (10) contemporary groups; (11) baseline equivalence of groups; (12) adequate statistical analyses.

## Data Availability

Data are contained within the article.
